# Hepatitis C virus E2 protein involve in insulin resistance through an impairment of Akt/PKB and GSK3β signaling in hepatocytes

**DOI:** 10.1186/1471-230X-12-74

**Published:** 2012-06-21

**Authors:** Ming-Ju Hsieh, Kuang-Ping Lan, Hao-Yu Liu, Xiao-Zong Zhang, Yaw-Feng Lin, Tzy-Yen Chen, Hui-Ling Chiou

**Affiliations:** 1 School of Medical Laboratory and Biotechnology, Chung Shan Medical University, Taichung, Taiwan; 2 Department of Nursing, Kaomei College of Health Care & Management, Kaohsiung, Taiwan; 3 Department of Laboratory, CiShan Hospital, Department of Health, Kaohsiung, Taiwan; 4 Department of Physical Education, National Pingtung University of Education, Pingtung, Taiwan; 5 Department of Internal Medicine, Chung Shan Medical University Hospital, Taichung, Taiwan; 6 Department of Internal Medicine, School of Medicine, Chung Shan Medical University, Taichung, Taiwan; 7 Department of Clinical Laboratory, Chung Shan Medical University Hospital, Taichung, Taiwan

**Keywords:** Hepatitis C virus, E2 envelope protein, Type 2 diabetes mellitus, Insulin resistance, Insulin receptor substrate-1 (IRS-1), GSK3β

## Abstract

**Background:**

Hepatitis C virus (HCV) infection may cause liver diseases of various severities ranging from primary acute infection to life-threatening diseases, such as cirrhosis or hepatocellular carcinoma with poor prognosis. According to clinical findings, HCV infection may also lead to some extra-hepatic symptoms, including type 2 diabetes mellitus (DM). Since insulin resistance is the major etiology for type 2 DM and numerous evidences showed that HCV infection associated with insulin resistance, the involvement of E2 in the pathogenesis of type 2 DM and underlying mechanisms were investigated in this study.

****Methods**:**

Reverse transcription and real-time PCR, Western blot assay, Immunoprecipitation, Glucose uptake assay and analysis of cellular glycogen content.

**Results:**

Results showed that E2 influenced on protein levels of insulin receptor substrate-1 (IRS-1) and impaired insulin-induced Ser308 phosphorylation of Akt/PKB and Ser9 phosphorylation of GSK3β in Huh7 cells, leading to an inhibition of glucose uptake and glycogen synthesis, respectively, and eventually insulin resistance.

**Conclusions:**

Therefore, HCV E2 protein indeed involved in the pathogenesis of type 2 DM by inducing insulin resistance.

## Background

An infection of hepatitis C virus may lead to serious liver diseases including chronic hepatitis, cirrhosis and hepatocellular carcinoma with poor prognosis [1-4]. HCV is an enveloped virus with a single-stranded positive-sense RNA genome of about 9.6 kb encoding 10 different proteins, including 3 structural proteins core, E1, E2 and 7 nonstructural proteins p7, NS2, NS3, NS4A, NS4B, NS5A and NS5B [5-7].

Besides causing liver diseases, HCV may lead to some extra-hepatic complications including type 2 diabetes mellitus (T2DM) [8] based on some epidemiological evidences. Furthermore, regardless of cirrhosis, the prevalence of T2DM of patients with HCV infection was much higher than that of patients with HBV infection [9-11]. It clearly indicated that HCV may somehow involve in the pathogenesis of T2DM, which causes hyperglycemia that involving multiple factors, especially insulin resistance [12]. Individuals with insulin resistance may produce sufficient insulin, but can’t effectively reduce blood sugar level because of resisting the function of the insulin, which mainly involves an impairment of insulin signaling pathway. Insulin receptor (IR) is a receptor with tyrosine kinase located on plasma membrane which contains two subunits, α and β subunits [13,14]. The tyrosine residues of IRS-1 also can be phosphorylated to turn on the insulin signaling pathway. The phosphorylated IRS-1 actives phosphatidylinositol 3-kinase (PI3K) that promote Phosphatidylinositol 4, 5-bisphosphate (PIP_2_) conversion into phosphatidylinositol-3,4,5-triphosphate (PIP_3_) following leads to Akt/PKB (protein kinase B) undergoes serine/threonine phosphorylation at the residues of Ser473 and Thr308 [15,16]. Finally, it promotes glucose transporter-4 (GLUT-4) translocation to plasma membrane to enhance glucose uptake [17,18]. According to the mechanism of insulin resistance, impairment of activation of Akt/PKB or GSK3 is the key role which can inhibit glucose uptake or glycogen synthesis, respectively.

Evidences indicated that cells expressing HCV proteins possess impaired insulin signaling [19]. Among HCV proteins, core protein was the most investigated target with extensive data accumulated during recent years [20-22]. Studies suggested that core protein might inhibit insulin signaling through increasing the expression of suppressors of cytokine signaling 3 (SOCS3) [20] or upregulating serine phosphorylation of IRS-1 by activation of c-Jun N-terminal kinase (JNK) [21]. The resultant event is the impairment of IRS-1 function and the downstream signaling. Such core protein-related mechanisms seem genotype-specific [22]. However, the involvement of HCV proteins other than core protein have not been investigated. Among them, E2 is mainly responsible for viral entry and plays an important role for virus infection. Nevertheless, the involvement of E2 in type 2 DM has not been investigated. Some evidences suggested HCV E2 protein could activate extracellular signal-regulated kinase (ERK) signaling which interact with cell surface CD81 [23-25]. ERK is one of the IRS-1 kinases which can upregulate serine phosphorylation of IRS-1 following an inhibition of IRS-1 function and eventually leading to insulin resistance [26]. Therefore, this study was conducted to understand the ability of HCV E2 for the induction of insulin resistance and the role of HCV E2 protein in insulin signaling pathway in hepatocytes.

## Methods

### Antibodies and reagent

Human recombinant insulin was purchased from Roche. SOCS3 antibody was from Santa Cruz Biotechnology. IRS-1, insulin receptor-beta (IRβ), phosphor-Akt (Ser473), ubiquitin, GSK3β and phosphor-GSK3β (Ser9) antibodies were from Cell Signaling Technology. phosphor-Akt (Thr308) antibodies was from Novus Biological. Akt/PKB and β-actin antibodies were from BD Biosciences. HCV E2 protein genotype 1b antibody was from Abcam. Peroxidase-conjugated goat anti-rabbit and goat anti-mouse secondary antibodies were from Jackson Immunoresearch Laboratories.

### Cell culture and transfection

Huh7, a human hepatoma cell line, obtained from the Food Industry Research and Development Institute (Hsinchu, Taiwan), were cultured in Dulbecco’s modified Eagle’s medium (DMEM) (Gibco BRL, Grand Island, NY, USA) supplemented with 10% fetal calf serum (FCS), 1 mM glutamine, 1% penicillin/streptomycin, 1.5 g/L sodium bicarbonate, and 1 mM sodium pyruvate (Sigma, St. Louis, Mo, USA). For transfection, 2 × 10^5^ cells were seeded on a culture dish of 6 cm on the day prior to transfection and then transfected with FLAG-CMV2 or FLAG-E2 plasmid [27] using TurboFect^TM^*in vitro* transfection reagent (Fermentas Life Sciences) according to the manufacturer’s instructions. After an overnight incubation, western blot was conducted to detect E2 protein level to ensure the successful expression. For insulin stimulation, cells were incubated with serum-free DMEM for 16 hours followed by a treatment with 100 nM insulin for an indicated time.

### Reverse transcription and real-time PCR

Total cellular RNA was extracted using TriSolution (GeneMark) and phenol/chloroform method. After being precipitated with isopropanol, 2 μg of total cellular RNAs were subjected to cDNA synthesis by MMLV reverse transcriptase (Promega) and oligo-dT primer according to the manufacturer’s instructions. For the quantification of human IRS-1 (Hs00178563_m1), real-time PCR with TaqMan® Fast Universal PCR Master Mix and TaqMan® specific primer with MGB probe (all from Applied Biosystems) were conducted with a normalization with human GAPDH (Hs99999905_m1). Real-time PCR was also conducted for insulin receptor (IR) with FastStrat Universal SYBR Green Master (Roche) and primers (forward 5′-CGGCCAGAGGCTGAGAATAAT-3′, reverse 5′-CGCCATCTGAATCA TCTCTTGA-3). All real-time PCR assays were performed on StepOne^TM^ Real-Time PCR System (Applied Biosystems) and the Ct value was analyzed by StepOne^TM^ Software v2.0.

### Western blot assay

Cells were harvested and lysed using RIPA buffer (50 mM Tris–HCl pH8.0, 0.1% SDS, 1% NP40, 150 mM NaCl, 20% glycerol, 2 mM dithothreitol, 0.5% deoxycholate acid) containing protease inhibitors and phosphates inhibitors. The cell lysates were separated in polyacrylamide gels and transferred onto polyinylidene fluoride membrane. Afterwards, membranes were incubated with blocking buffer (5% non-fat milk in TBST (TBS buffer with 0.1% Tween-20)) for 1 hour, and incubated with specific primary antibody overnight in 4 °C. After washing with TBST for 3 times, members were incubated with an appropriate peroxidase-conjugated secondary antibody for 1 hour in room temperature followed by TBST washing for 3 times. Signal was developed by chemiluminescent HRP substrate (Millipore) and detected by LAS-1000 Luminescent Image Analyzer (FUJIFILN). Relative photographic density was quantitated by scanning the photographic negatives on a gel documentation and analysis system (Alpha Imager 2000, Alpha Innotech Corporation).

### Immunoprecipitation

E2-transfected and un-transfected Huh7 cells were lysed on ice for 20 minutes in RIPA buffer. After centrifugation, supernatant was incubated with IRS-1 antibody at 4 °C for overnight, followed by incubation with protein A/G-PLUS-agarose at 4 °C for 1 hour. Immunocomplexes were washed After being washed for 3 times in RIPA buffer, the reaction was terminated by adding 5× SDS sample buffer and then subjected to western analysis.

### Glucose uptake assay

Cells cultured in 24-well plates were deprived of serum by incubation in serum-free medium for 16 hours. The cells were then washed with KRH (−) glucose (12 mM Hepes, 121 mM NaCl, 4.9 mM KCl, 1.2 mM MgSO_4_, 0.33 mM CaCl_2_, pH 7.4). In brief, cells were initiated by addition 225 μL of working solution of insulin in KRH (−) glucose into each well for 13 minutes. At the end of incubation, 1.25 μL of cytochalasin B stock solution was added into wells while 1.25 μL of 100% DMSO was added to other wells followed by a gentle shaking for 2 minutes. Glucose uptake was initiated by an addition of 25 μl of reaction solution (KRH (−) containing 0.04 mM, 2-deoxy-d-[1,2-^3^ H] glucose) to each well. After 5 minutes, transport was terminated by washing the cells with ice-cold KRH (+) glucose (KRH (−) glucose containing 25 mM d-(+)-Glucose). The cells were solubilized by 0.1% sodium dodecyl sulfate, and the incorporated radioactivity was measured by liquid scintillation counter.

### Analysis of cellular glycogen content

To measure the content of glycogen, Huh7 cells were seeded at a density of 2 × 10^5^ cells/dish. After an overnight incubation, cells were transfected with or without HCV E2 expression plasmid. After being cultured in serum-free medium overnight, cells were cultured in DMEM containing 100 nM insulin for 9 hours. Cells were washed to remove extracellular glucose and homogenized with 200 μl dH_2_O on ice. Homogenates were boiled for 5 minutes and spin at 13000 rpm for 5 minutes. The final supernatant was then subjected to glycogen assay kit (Biovision).

### Statistical analysis

Statistical significances of difference throughout this study were calculated by One-Way ANOVA test using SPSS software. P value less than 0.05 was regarded as statistically significant.

## Result

### HCV E2 expression had an influence on IRS-1 protein level

**Figure 1:**
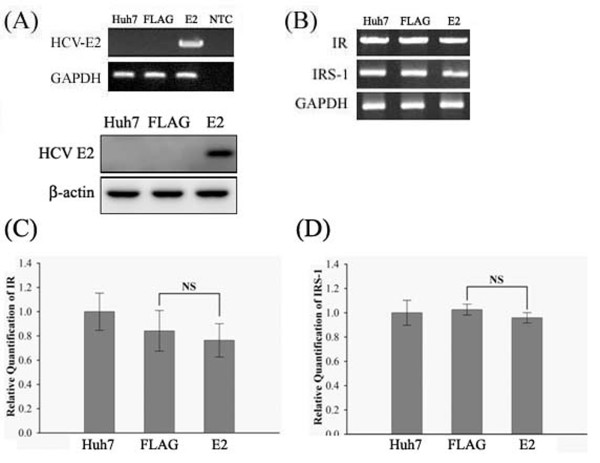
**IR and IRS-1 mRNA levels were unchanged with HCV E2 protein expression.** Huh7 cells were transfected with FLAG-CMV2 or FLAG-E2 for 24 hours. After the confirmation of successful E2 expression (**A**), cells were subjected to semi-quantitative PCR (**B**) and RT-PCR (**C** &**D**) to show IR and IRS-1mRNA expression levels were not changed by E2 expression. The error bars represented standard deviation from triplicate experiments. NS means not significant.

**Figure 2:**
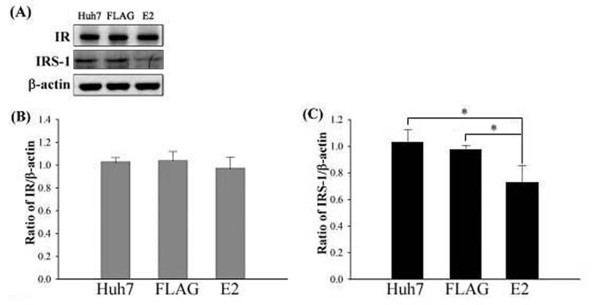
**IR and IRS-1 protein levels while HCV E2 protein expressed in Huh7.** Huh7 cells were transfected with FLAG-CMV2 or FLAG-E2 for 24 hours and then subjected to western blotting for IR and IRS-1 with β-actin acting as internal control (**A**). Relative photographic density was quantified and the results were shown as histograms mean relative IR (**B**) and IRS-1 (**C**) levels after being normalized by β-actin. While the protein level of IR was unchanged, that of IRS-1 protein expression was decreased by HCV E2 expression. The error bars represented standard deviation from triplicate experiments. * indicates p<0.05.

Upon the stimulation of insulin, activation of IR and IRS-1 are the first key steps for glucose metabolisms. An altered IR expression or tyrosine phosphorylation will lead to insulin resistance [28,29]. Therefore, the impact of HCV E2 protein on IR or IRS-1 expression was investigated. After a transfection of FLAG-E2 expression plasmid RT-PCR and western blot assays were conducted to ensure a successful expression of E2 protein in Huh7 cells (Figure 1A). Then, IR and IRS-1 expression levels were detected by semi-quantitative RT-PCR, real-time RT-PCR and western blot. Results of real-time RT-PCR suggested that HCV E2 protein has no influence on mRNA levels of IR and IRS-1 (Figure 1B1C1D). However, IRS-1 protein level was significantly reduced responding to the expression of HCV E2, as shown by results of western blot (Figure 2).

### The expression levels of SOCS3 is increased in HCV E2-transfected Huh7

**Figure 3:**
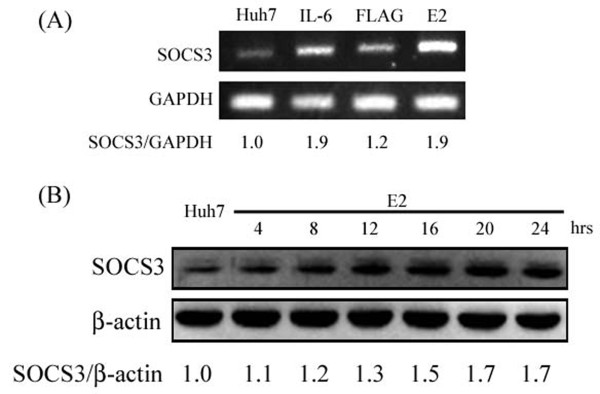
**HCV E2 induce SOCS3 mRNA and protein levels in Huh7.** Huh7 cells were transfected with FLAG-CMV2 or FLAG-E2 for 24 hours and then subjected to semi-quantitative PCR for SOCS3 expression with GAPDH being an internal control (**A**). For western blotting, cell lysate samples were prepared from Huh7 cells transfected with FLAG-E2 for the indicated time and then subjected to western blotting for SOCS3 with β-actin and IL-6 treatment (100 ng/ml for 30 minutes) being an internal control and positive control, respectively. Results in (**B**) indicated that the expression levels of SOCS3 were increased by E2 expression.

SOCS3 is known to be a negative regulator for cytokine signaling such as interleukin-6, growth hormone, and interferon-α [30] and also an interfere factor for insulin signaling by promoting ubiquitin-mediated degradation of IRS proteins [31,32]. Therefore, the expression levels of SOCS3 in HCV E2-transfected Huh7 cells were determined by semi-quantitative RT-PCR and western blot. Results shown in Figure 3 indicated expression of HCV E2 led to a dose-dependent increase of SOCS3 expression at both transcriptional and translational level.

### Ubiquitination is involved in the E2-induced reduction of IRS-1 expression

**Figure 4:**
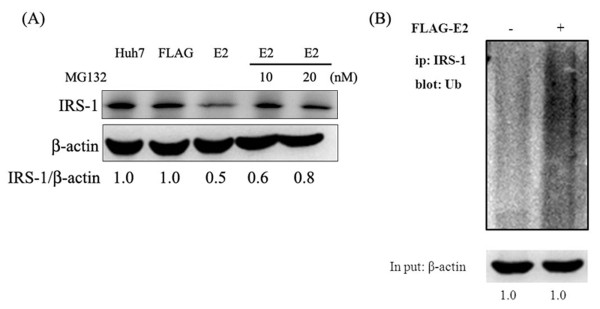
**HCV E2-caused down-regulation of IRS-1 may be through an ubiquitination.** In the absence or presence of MG132 (10 and 20 nM), Huh7 cells were transfected with FLAG-CMV2 or FLAG-E2 for 24 hours and then subjected to immunoblotting with the indicated antibodies (**A**). Results indicated that MG132, proteosomal proteolysis inhibitor, may restore the E2-related down-regulation of IRS1. Furthermore, cell lysates were subjected to immunoprecipitation with IRS-1 followed by immunoblotting with anti-ubiquitin antibody. Results in (**B**) indicated that HCV E2 caused an accumulation of ubiquitin-conjugated IRS-1.

To investigate the underlying mechanism for E2-induced reduction of IRS1 expression, E2-transfected cells were subjected to a treatment with MG132, a proteosomal proteolysis inhibitor. Results of western blot revealed that a treatment with MG132 may restore the expression levels of IRS1 in E2-transfected Huh7 cells (Figure 4A). Furthermore, immunoprecipitation with anti-IRS-1 and immunoblotting with anti-ubiquitin antibodies were conducted to show that the presence of HCV E2 caused an accumulation of ubiquitin-conjugated IRS-1, as shown in Figure 4B.

### Insulin-induced threonine phosphorylation of akt and serine phosphorylation of GSK3β is abolished in E2-transfected Huh7 cells

**Figure 5:**
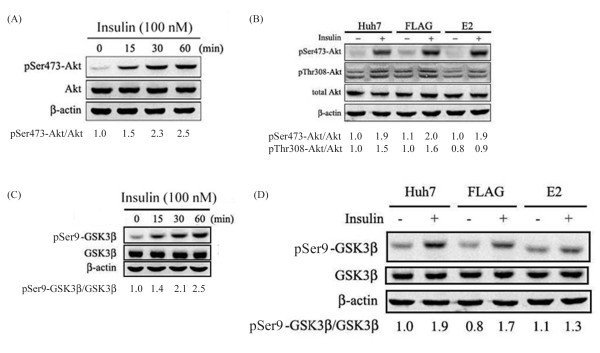
**Insulin-induced phosphorylation of Akt and GSK3β was inhibited by E2 protein.** After being transfected with FLAG-CMV2 or FLAG-E2 for 24 hours and a serum-free incubation 16 hours, cells were subjected to a insulin stimulation for an indicated time period (0, 15, 30 and 60 minutes) followed by western blotting with indicated antibodies to reveal a time-dependent increase of phosphorylation of Akt (**A**) and GSK3β (**C**). Furthermore, transfected or untransfected cells were treated insulin for 30 minutes and then subjected to western blotting to detect Ser473 and Thr308 phosphorylation of Akt (**B**) serine phosphorylation of GSK3β (**D**). Results suggested that an expression of E2 inhibited insulin-induced Thr308 phosphorylation of Akt and GSK3β phosphorylation.

According to a previous research, IR and IRS-1 protein expression levels in liver samples from patients with HCV infection were not altered, while insulin-induced phosphorylation of IRS-1, PI3K and Akt were impaired [33]. Therefore, the effect of HCV E2 protein on insulin-induced phosphorylation of Akt was investigated. Results from a time course experiment for condition determination indicated that Ser473 phosphorylation level of Akt was increased to 2.5 folds in Huh7 cells underwent an insulin treatment for 15 minutes, as compared to that of untreated cells (Figure 5A). A same extent of insulin-induced increase in Ser473 phosphorylation of Akt was seen in FLAG-transfected and E2-transfected Huh7 cells, indicating that E2 protein has no effect on insulin-induced Ser473 phosphorylation of Akt. While insulin-induced Thr308 phosphorylation of Akt is down-regulated in E2-transfected Huh7 cell suggesting that HCV E2 protein-induced insulin resistance may occur via a downregulation of Thr308 phosphorylation of Akt, which may serve as a signature molecule in HCV E2-mediated insulin resistance (Figure 5B).

It is known that glycogen synthase kinase 3 (GSK3) is key component of glycogen synthesis that can inhibit the activity of glycogen synthase (GS). Furthermore, activated Akt/PKB can induce serine phosphorylation of GSK3 (Ser21 for GSK3α; Ser9 for GSK3β) to inactivate the kinase activity, which subsequently lead to an activation of GS and then increased glycogen synthesis [34,35]. To assess the influence of HCV E2 protein on insulin-induced GSK3β phosphorylation. Western blottings were conducted to show that a 15-minute treatment of insulin may induce serine phosphorylation of GSK3β in both un-transfected and FLAG-transfected Huh7 cells (Figure 5C). Furthermore, such insulin-induced GSK3β phosphorylation was inhibited by an expression of E2, as compared to that of untransfected- and FLAG-transfected Huh7 cells (Figure 5D).

### Insulin-induced cellular glucose and glycogen contents were reduced in response to the expression of E2 protein

**Figure 6:**
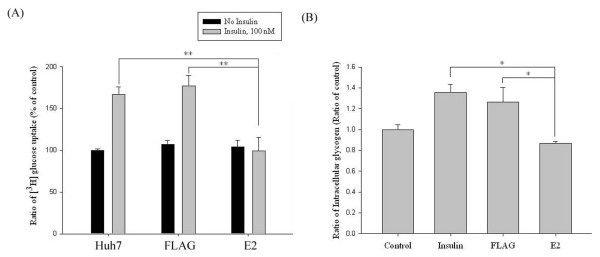
**Insulin-induced glucose uptake and glycogen synthesis were both decreased by an expression of E2 protein.** After being transfected with FLAG-CMV2 or FLAG-E2 for 24 hours and a serum-free incubation 16 hours, cells were subjected to insulin stimulation for 13 minutes and then glucose uptake analysis with 2-deoxy-d-[1,2-^3^ H] glucose. [^3^ H] glucose uptake levels were quantified with liquid scintillation counter and presented as a percentage to that of control (**A**). For glycogen synthesis analysis, the insulin stimulation was carried out for 9 hours before cells were subjected to glycogen assay kit (**B**). These results clearly indicated that E2 protein can inhibit insulin-induced cellular glucose uptake and glycogen synthesis. The error bars represented standard deviation from triplicate experiments. * indicates p<0.05 and ** indicates p<0.01.

To investigate whether HCV E2 protein had the inhibitory ability for insulin-induced cellular glucose uptake, 2-deoxygluose uptake levels were determined. Compared to that in untransfected and FLAG-transfected Huh7 cells, insulin-induced uptake levels in E2-transfected Huh7 cells were significantly suppressed by about 66-77% (Figure 6A). Meanwhile, intracellular glycogen levels were significantly increased by a 9-hour treatment of insulin, while such enhancement was decreased for about 45% in E2-transfected Huh7 cells, as compared with those untransfected or FLAG-transfected cells (Figure 6B). These results suggest that E2 protein can inhibit insulin-induced cellular glucose uptake and glycogen synthesis.

## Discussion

Chronic viral hepatitis, mostly caused by hepatitis B virus (HBV) or hepatitis C virus (HCV), has been an important medical global problem with serious consequences including cirrhosis and hepatocellular carcinoma. Furthermore, several epidemiological studies suggested that HCV infection, but not HBV, may contribute the incidence of type 2 diabetes mellitus (T2DM). Patients with chronic HCV infection had a higher ratio of about 1.8 to 2.5 folds for T2DM, as compared with that of patients with HBV infection and controls [9-11]. Among multiple factors involved in the occurrence of T2DM, insulin resistance is the major one. Therefore, detailed mechanisms involved in the relationship between HCV infection and insulin resistance is worthy to be investigated.

Regarding the involvement of HCV proteins in insulin resistance, results from intensive studies indicated that HCV core protein could cause IRS-1 and IRS-2 degradation through proteasome ubiquitination by inducing SOCS3 expression, and induce Ser312 phosphorylation of IRS-1 to impede insulin signaling [20,21]. Furthermore, study conducted by Pazienza et al. revealed that the mechanism of IRS-1 signaling impairment by HCV core protein seems to be genotype-specific. For genotype 3a, core protein causes IRS-1 degradation through SOCS7 upregulation and PPAR-γ downregulation, while in case of genotype 1b, core protein leads to impaired IRS-1 signaling by activation of mTOR activity [22]. In addition to core protein, the involvement of other viral proteins should be investigated. Our previous studies showed that HCV E2 protein can influence cell apoptosis [27] and fibrosis by interfering multiple signaling pathways, and therefore, E2 protein is extremely possible to involve in the insulin signaling pathway. Based on this assumption, whether HCV E2 protein has the ability of induction of insulin resistance and relevant molecular mechanisms were investigated in this study.

With a Huh7 cell line expressing HCV E2 protein, our results showed that HCV E2 protein can cause IRS-1 protein degradation while the mRNA levels of IR and IRS-1 were both not altered. Since these two components are key components in the upstream of insulin signaling pathway, such impairment may lead to insulin resistance [20,28,29]. Previous studies show that HCV core protein may cause IRS-1 and IRS-2 degradation through proteasome ubiquitination by inducing SOCS3 expression [20]. Our results also confirmed that HCV E2 protein can enhance mRNA and protein expression levels of SOCS3 (Figure 3) and E2-induced IRS-1 down-regulation was restored by a treatment of MG132. These results clearly indicated that HCV E2 protein can stimulate SOCS3 expression to lead to IRS-1 degradation though proteasome ubiquitination. Based on other reports, it was indicated that HCV infection may also impair the insulin-induced IRS-1, PI3K and Akt phosphorylation that lead to insulin resistance [17,33]. Previous studies shown that phosphorylation of Akt at Ser473 appears to precede phosphorylation by PDK1 at Thr308, both sites are separated by 165 amino acids [36]. Inhibition of Thr308, but not Ser473 phosphorylation of Akt has been suggested as a basis for decreased insulin-stimulated glucose transport [37,38]. In a recent study, it was shown that HCV-induced down-regulation of GLUT2 expression and up-regulation of gluconeogenesis [39]. This suggests that high concentration of glucose in HCV-infected hepatocytes, although interpretation of their data should be cautioned because they used a HCV J6/JFH1-infected cells. Likewise, our results showed that HCV E2 protein can impair insulin-induced Akt Thr308 phosphorylation (Figure 5B), and subsequently inhibit glucose uptake (Figure 6A). Other than an enhancement of glucose uptake, Akt phosphorylation also involves in GSK3-dependent pathway which responsible for glycogen synthesis and it was reported that HCV-induced insulin resistance may be through impairment of insulin-induced GSK3β phosphorylation [19]. As results shown in Figure 5D, such insulin-induced GSK3β phosphorylation was reversed by an expression of HCV E2, followed by an inhibition of glycogen synthesis (Figure 6B) and lead to insulin resistance. Since GSK3 involves in a lot of cellular processes including glycogen synthesis, the appearance of increased GSK3β phosphorylation in Huh7-FLAG without insulin treatment could be related to cell transfection process, but not cell starvation before insulin stimulation (data not shown).

## **Conclusions**

Based on abovementioned results, we believe that decreased IRS-1 expression and induced SOCS3 expression are involved in HCV E2 protein-induced insulin resistance through an impairment of Akt/PKB and GSK3β signaling, which inhibit glucose uptake and glycogen synthesis, respectively.

## Competing interests

The authors declare that they have no competing interests.

## Authors’ contributions

M-JH has made substantive intellectual contributions to a published study and drafted the manuscript. K-PL, HL, X-ZZ and Y-FL have made substantial contributions to conception and design, or acquisition of data, or analysis and interpretation of data. T-YC and H-LC conceived of the study, and participated in its design and coordination and helped to draft the manuscript. All authors read and approved the final manuscript. This study will be presented in the 6th Ditan International Conference on Infectious Diseases (DICID) to be held in Beijing, China i, UAE, from July 12 to 15, 2012.

## Pre-publication history

The pre-publication history for this paper can be accessed here:

http://www.biomedcentral.com/1471-230X/12/74/prepub
